# Method for Estimating Road Impulse Ahead of Vehicles in Urban Environment with Microelectromechanical System Three-Dimensional Sensor

**DOI:** 10.3390/s24041192

**Published:** 2024-02-11

**Authors:** Shijie Zhao, Minghao Wang, Pengyu Wang, Yang Wang, Konghui Guo

**Affiliations:** State Key Laboratory of Automotive Simulation and Control, Jilin University, Changchun 130025, China; zhaosj17@mails.jlu.edu.cn (S.Z.); wangmh20@mails.jlu.edu.cn (M.W.); yangwang@jlu.edu.cn (Y.W.); guokh@jlu.edu.cn (K.G.)

**Keywords:** road impulse features, LiDAR error model, pose estimation, MEMS LiDAR

## Abstract

Most automated vehicles (AVs) are equipped with abundant sensors, which enable AVs to improve ride comfort by sensing road elevation, such as speed bumps. This paper proposes a method for estimating the road impulse features ahead of vehicles in urban environments with microelectromechanical system (MEMS) light detection and ranging (LiDAR). The proposed method deploys a real-time estimation of the vehicle pose to solve the problem of sparse sampling of the LiDAR. Considering the LiDAR error model, the proposed method builds the grid height measurement model by maximum likelihood estimation. Moreover, it incorporates height measurements with the LiDAR error model by the Kalman filter and introduces motion uncertainty to form an elevation weight method by confidence eclipse. In addition, a gate strategy based on the Mahalanobis distance is integrated to handle the sharp changes in elevation. The proposed method is tested in the urban environment. The results demonstrate the effectiveness of our method.

## 1. Introduction

Safety, reliability, comfort, and economy have become the key factors for the development of high-quality automated vehicles (AVs). The main source of vertical input for an AV is road unevenness [1]. The unevenness of the road surface directly affects the ride comfort, smoothness, and operation stability. Due to the diversity of functional requirements and the complexity of the driving environment, AVs often have many heterogeneous sensors [2], such as light detection and ranging (LiDAR), camera, inertial measurement unit, etc. The road impulse features, such as speed bumps in front of the vehicle, can be sensed by relying on these powerful sensors. The semi-active or active suspension of the vehicle can be adjusted in advance before the vehicle reaches the target area. This fundamentally solves the problem of time delay in traditional control methods and improves the ride comfort of the car.

AVs require knowledge of the surroundings to safely and efficiently interact with the environment. The LiDAR is one of the most popular sensors in the field of autonomous driving due to its long range and high accuracy in 3D measurement. Existing approaches were mainly developed for mechanical LiDAR sensors, which collect the surrounding information by spinning a high-frequency laser array [3]. However, due to its high cost and weight, the mechanical LiDAR is difficult to implement on AVs as a mass production solution. In the last few years, the solid-state LiDAR has gained more interest due to its cost-effectiveness and lightweight. The microelectromechanical system (MEMS) solid-state LiDAR is a system that is built entirely on a silicon chip with no moving parts involved [4]. Therefore, it has advantages in size and weight compared to the mechanical LiDAR. Moreover, the MEMS solid-state LiDAR is resistant to vibrations by removing the rotating mechanical structure, which enhances its reliability and durability [3]. To illustrate the difference between the two LiDARs, we used Velodyne HDL-64E and RoboSense RS-LiDAR-M1, for example. The specifications can be found in Table 1.

Perceiving the road impulse information is a vital capacity for semi-active or active suspension preview control. This task is usually accomplished by building a map of the road using acquired sensor data. Several mapping methods have been proposed to build a dense map based on trajectory estimation, such as an occupancy grid map and elevation map [5]. An elevation map replaces the binary information in the occupied grid map with elevation so that it is widely applied in outdoor environments. Generally, a global elevation map is built offline when the trajectory is estimated by simultaneous localization and mapping (SLAM). However, in scenarios where no prior map is available, the trajectory is estimated and corrected by SLAM online [6]. It means that we have to not only save all sensor data but also overcome the sensitivity to environmental conditions. For suspension preview control, we focused on the mapping accuracy of observed regions in front of the AVs rather than global mapping accuracy. Moreover, this mapping method can better achieve real-time map updates. Zhao et al. [7] and Wang et al. [8] deployed the local mapping framework to extract the preview elevation of roads based on LiDAR, IMU, and GPS, which achieved real-time and accurate estimation to some extent. Although the aforementioned methods show a good performance, the relatively low vehicle speed allows sufficient time for estimation. Therefore, we constructed a real-time local road elevation estimation. The main idea is to implement a local map through grid height based on the sensor error model and motion uncertainty. The proposed method leverages lightweight pose estimation and grid height models to reduce computational costs while considering uncertainty in the map update to improve accuracy. Our contributions are summarized as follows: (1) a real-time estimation algorithm of the vehicle pose by a MEMS LiDAR with a small FOV; (2) a grid height model based on the LiDAR error model to aggregate multiple points within a grid; (3) by deriving error propagation to align the maps of consecutive frames, lightweight local map updates are achieved; and (4) a gate strategy based on the Mahalanobis distance to deal with the sharp changes in elevation.

This paper is organized as follows: Section 2 reviews the related works on existing LiDAR elevation estimation approaches. Section 3 describes the details of the proposed real-time local road elevation estimation method. Section 4 shows experimental results and comparisons with existing works, followed by a conclusion in Section 5.

## 2. Related Work

A vehicle’s surroundings can be geometrically modeled by constructing representations of the underlying terrain surface using range sensor data. These range sensors produce sparse point cloud representations, which must then be converted into continuous or piecewise structures to use [9]. Many methods have been proposed to describe the terrain under the vehicle. The occupancy grid map [10] proposed by Elfes is the most common one. The occupancy grid map describes the environment as some regular grids of a specific size. And the value of each grid represents the probability that the grid is occupied. The above concept of a 2D occupancy grid map is intuitively extended to a 3D occupancy grid map. Kudriashov et al. [11] proposed a method for constructing a 3D occupancy grid map of unknown terrain by LiDAR. The construction of the 3D map and the pose estimation of the system are carried out simultaneously using the extended Kalman filter and other probabilistic methods. Three-dimensional occupancy grid maps provide abundant information and less ambiguity [12]. Another idea is to store information about 3D voxels in the octree, such as OctoMap [13]. The voxel size is a design parameter that influences memory usage, computation time, and, more importantly, the accuracy of the map. However, the voxels are distributed discretely, and we lose information about details inside each voxel. The space inside each cell is better represented in the normal distributions transform occupancy map [14]. And it was successfully applied on a large scale [15] and for highly dynamic environments [14]. Another 3D geometric description of the terrain is signed distance fields (SDF) or truncated signed distance Fields (TSDF). These methods map the distance to the nearest occupied cell rather than storing the entire volumetric representation of the environment [16]. Canelhas et al. utilize SDF to represent the alignment error and estimate the motion of the camera [17]. Sommer et al. [18] proposed gradient-SDF to describe 3D geometry that combines the advantages of implicit and explicit representations. Grinvald et al. [19] extended the TSDF model using information about object categories. Lang et al. [20] proposed a monocular SLAM system with direct TSDF mapping based on a sparse voxelized recurrent network. The direct TSDF mapping achieves simultaneous estimation of pose and map using features.

However, the 3D occupancy grid map will cause extremely high computational costs. It fails to strike a balance between resolution and computation time, which makes the method unavailable in real-time scenarios. As a compromise, the 2.5D occupancy grid map method has been proposed in recent years. Each of the grid values is redefined as the height of the grid. Fankhauser et al. [21,22] proposed a 2.5D grid map-building method considering the uncertainty of robot motion. Souza et al. [23] described the occupancy-elevation grid mapping method, where each cell represents the probability, height, and variance of occupancy. Zhou et al. [24] employed the preemptive RANSAC algorithm to extract planes from the terrain height information within the voxel grid. It enables the estimation of parameters such as height and depth in structured environments. Although the above methods achieved real-time mapping in some applications, highly accurate estimation is essential to accomplish reliable suspension preview control. Several works have introduced neural networks [25,26,27,28,29,30] to generate dense elevation maps. The neural networks effectively eliminate the noises and generate rational features in the occluded regions, which enhances the robustness and accuracy of methods in different environments. But, it is hard to implement real-time suspension preview control.

## 3. Method

**Figure 1:**
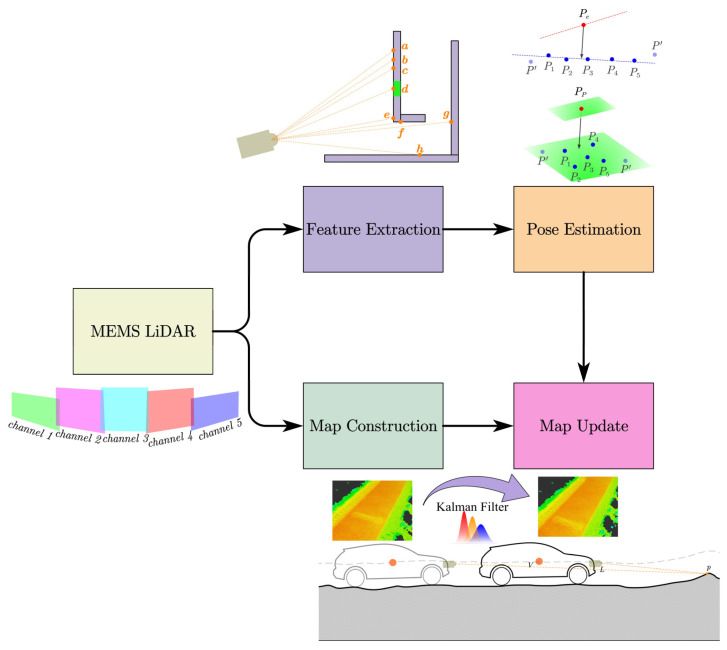
Framework of the proposed method.

The framework of our proposed method is illustrated in Figure 1. The arrows in the Figure 1 represent the data flow between different parts. In the feature extraction part, we selected feature points that can describe all MEMS LiDAR points. This function reduces the amount of data and speeds up processing. And in the pose estimation part, we utilized scan-to-map to estimate the vehicle pose. In addition, in the map construction part, all MEMS LiDAR points are mapped to the corresponding grid. When multiple points were projected into one grid, we applied the maximum-likelihood estimation to fuse the elevation at that grid. In the map update part, the consecutive frame data are accumulated by pose estimation to deal with the problem of LiDAR sparse sampling points. And the elevation map algorithm that incorporates range measurements with a sensor error model by Kalman filter was used.

### 3.1. Vehicle Pose Estimate

#### 3.1.1. Feature Extraction

The pose estimation is the bridge between consecutive frame measurements. Therefore, the proposed method develops feature extraction and matching rules based on the characteristics of the MEMS solid-state LiDAR. The resolution of the MEMS solid-state LiDAR is higher than the mechanical LiDAR. As mentioned above, RS-LiDAR-M1 scans 125×126×5 raw points in each frame, which is a heavy burden for the pose estimator. It is not only slow but also susceptible to noise to match the raw point clouds directly. Therefore, we down-sampled the raw point cloud through a voxel grid filter [31]. And we further processed the point cloud data in the feature space. Inspired by LOAM [32], we extracted features from the down-sampled point cloud data to transform the point cloud into features with obvious physical significance to improve the quality of registration. At the same time, feature extraction achieves mapping from high-dimensional space to low-dimensional space, making the matching speed meet real-time requirements.

Specifically, plane features and edge features were extracted from point clouds. And we divided them according to the local smoothness. The local smoothness f is described as

(1) $$f=\frac{1}{|S|\cdot \left\|P_{\left(k,i\right)}^{L}\right\|}\left\|\sum_{j\in S,j\neq i}\left(P_{\left(k,i\right)}^{L}-P_{\left(k,j\right)}^{L}\right)\right\|$$

where $P_{\left(k,i\right)}^{L}$ represents the coordinate of the point i in the kth frame in the LiDAR coordinate system. And $P_{\left(k,j\right)}^{L}$ represents the coordinate of the point j in the kth frame in the LiDAR coordinate system. S represents the set of continuous points from the same row in each frame of the LiDAR. And half of the points in S are on either side of $P_{\left(k,i\right)}^{L}$. In this paper, we set the size of S to 10, namely |S| is 10. A larger local smoothness f means the surrounding plane is curved, while a smaller local smoothness f means the surrounding plane is smooth.

**Figure 2:**
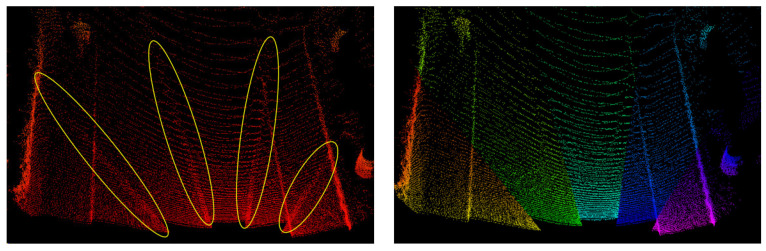
Illustration of comparison before and after removal.

In practice, due to the staggered scanning channels of RS-LiDAR-M1, the data of the five channels are processed separately. In addition, unlike the mechanical LiDAR, the RS LiDAR-M1 uses a spiral scanning trajectory for each channel. The scanning trajectory at the edge of the channel has overlap and large curvature, which is marked with yellow ellipses in Figure 2. And it does not conform to the assumption of smooth changes in adjacent points. Therefore, it is necessary to remove the spiral parts at the edge of the channel. The comparison before and after removal is shown in Figure 2. We have adopted different colors for each channel, which makes it easy to observe the reduction of the overlap between channels after removal.

In addition, in response to the small horizontal field FOV of RS LiDAR-M1, a reflection intensity smoothness $f_{I}$ is defined to alleviate its impact on feature point selection. The $f_{I}$ is described as

(2) $$f_{I}=\frac{1}{\left|S\right|}\left|\sum_{j\in S,j\neq i}\left(I(P_{\left(k,i\right)}^{L})-I(P_{\left(k,j\right)}^{L})\right)\right|$$

where $I(P_{\left(k,i\right)}^{L})$ stands for the reflection intensity of point $P_{\left(k,i\right)}^{L}$, and $I(P_{\left(k,j\right)}^{L})$ stand for the reflection intensity of point $P_{\left(k,j\right)}^{L}$. For any LiDAR point P=[x,y,z] with a reflectivity of *RE*, the reflection intensity is defined as $I(PC)=\frac{RE}{\sqrt{x^{2}+y^{2}+z^{2}}}$.

**Figure 3:**
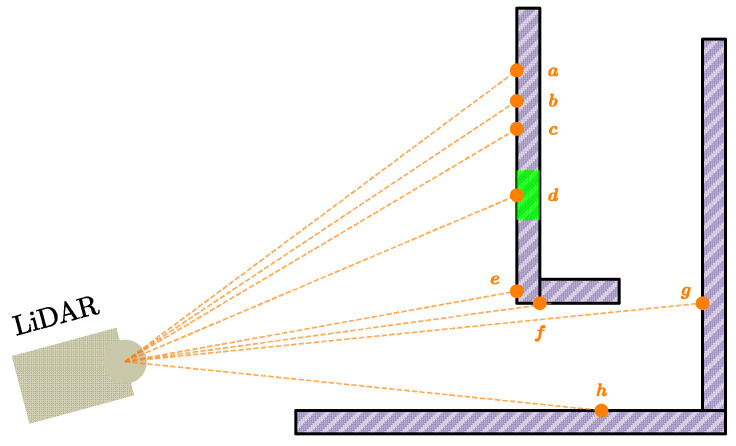
Illustration of the different types of the laser points.

The points in each scan channel are sorted based on the f value. Then, we selected the point with the maximum f value as the edge feature and the minimum f value as plane feature. Each scan channel can provide a maximum of 1 edge feature and 2 plane features. As shown in Figure 3, a–c are regarded as plane features, while e and f are regarded as edge features. In practice, we set the plane feature threshold to be less than 0.2 and the edge feature threshold to be greater than 0.5. A point i can be selected as an edge feature or a plane feature only if its f value is larger or smaller than a threshold and the number of selected points does not exceed the maximum.

Furthermore, to indicate the impact of the limited FOV of the solid-state LiDAR, we introduced reflectivity as another evaluation metric. Generally speaking, objects of different materials have different reflectivity. If the reflectivity of a point is very different from the surrounding points, there is a high probability that this point is the edge of the two materials. Therefore, we extended the concept of edge feature points, which regards such points as edge feature points. As shown in Figure 3, due to the green part being another material with different reflectivity, the point d is an edge feature point.

**Figure 4:**
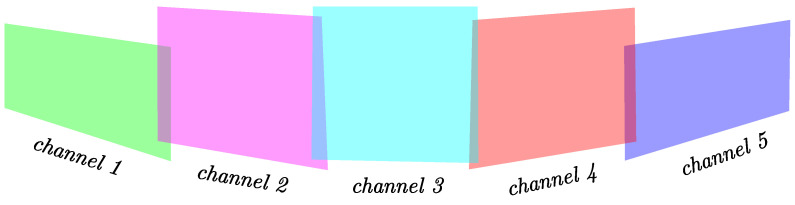
Demonstration of the RS-LiDAR-M1 scanning channel.

Considering the special measuring function of the MEMS solid-state LiDAR, the point cloud should be processed first. As is shown in Figure 4, the RS-LiDAR-M1 obtains data through five channels simultaneously. In other words, 5 points are received at the same time. There is a certain stagger in the vertical arrangement direction between the channel and the channel. And the FOV of each channel is not exactly the same. Since each channel is spiral scanning, there is a huge curvature at the edge of each channel, which causes a problem in feature extraction. Hence, we eliminated these edge points to increase the pose estimation accuracy. In addition, the LiDAR points that meet the following conditions also need to be eliminated:

Points that are on the boundary of occluded regions, such as point g in Figure 3. If the LiDAR moves a little to another place, these points are unobservable.

Points on local plane surfaces that are roughly parallel to the laser beams, such as point h in Figure 3. These points change dramatically with tiny movements of the LiDAR, which is unreliable.

Points with strange intensity. The intensity describes the strength of the received laser signal. Both too high and too low usually lead to weak confidence and accuracy.

#### 3.1.2. Pose Estimation

Pose estimation is the task of estimating the pose of the current moment relative to the previous moment based on historical scans. Estimation methods include scan-to-scan match and scan-to-map match [33]. A scan-to-scan match only relies on the point cloud data of the previous frame, which obtains a lower computational cost. However, the accuracy of this method is inevitably lower due to less information. Therefore, we implemented a scan-to-map match, which improves accuracy without excessive computational cost consumption.

In order to achieve a balance between performance and efficiency, we introduced a sliding window method to build the local map $M_{k}=\left\{P_{k-1},P_{k-2},\cdots ,P_{k-n}\right\}$, where n is the number of frames to build the local map. More specifically, the local map is divided into the edge map and the plane map. And the map in every time step is built by the K-D tree [34] to increase search efficiency.

As mentioned above, matching on raw point clouds is less efficient and sensitive to noise. Thus, we leveraged matching edge points and plane points in feature space. To find the nearest edge point from the local map, the edge point $p_{e}$ was projected to the local map by the following transformation:

(3) $${\hat{p}}_{e}=T_{k}\cdot p_{e}$$

where $T_{k}$ is the LiDAR pose in the kth frame and needs to be determined by the pose estimation.

**Figure 5:**
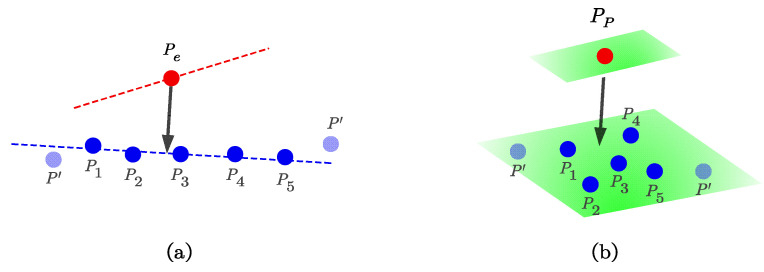
Illustration of the feature point residual. (**a**) Edge-to-edge residual. (**b**) Plane-to-plane residual.

In our work, we found five nearest points in the local edge map of each $p_{e}$, which is shown in Figure 5a. The arrow represents the corresponding relationship. To ensure that the five points are in a straight line such as P1–P5 located on blue dashed line in Figure 5a, we computed the mean μ and covariance matrix Σ formed by those five points. If the maximum eigenvalue of the matrix Σ is more than three times larger than the second largest eigenvalue, we believe that those five points are on a straight line. Then, the edge-to-edge residual is computed as the following:

(4) $$r_{e2c}=\frac{\left|\left(p_{e}-p_{5})\times (p_{e}-p_{1}\right)\right|}{p_{5}-p_{1}}$$

Similar to the edge residual, for each plane point $p_{p}$,we searched for the five nearest points in the local plane map such as P1–P5 located on green plane in Figure 5b, which is shown in Figure 5b. To ensure that five points are in the same plane, we also computed the mean μ and covariance matrix Σ formed by those five points. If the smallest eigenvalue of the matrix Σ is more than three times smaller than the second smallest eigenvalue, we believe that those five points are in the same plane. Then, the plane-to-plane residual is computed as the following:

(5) $$r_{p2p}=\frac{{\left(p_{p}-p_{1}\right)}^{T}\left(\left(p_{3}-p_{5})\times (p_{3}-p_{1}\right)\right)}{\left|\left(p_{3}-p_{5})\times (p_{3}-p_{1}\right)\right|}$$

Finally, the vehicle pose is estimated by minimizing the edge-to-edge residual and plane-to-plane residual:

(6) $$\arg\underset{T_{k}}{\min}\sum r_{e2c}+\sum r_{p2p}$$

### 3.2. Road Elevation Map Update by Microelectromechanical System Light Detection and Ranging Measurement

Stable and accurate road elevation estimation plays a crucial role in suspension preview control. This section constructs a vehicle-centric local elevation map. Firstly, an isotropic model is deployed to represent LiDAR error, where the parameters are obtained through experiments. Then, the maximum-likelihood estimation is used to aggregate measurements in the same grid. Secondly, considering the impact of motion uncertainty in map updates, the error propagation of pose estimation is derived. At the same time, the gate strategy based on the Mahalanobis distance is adopted to filter the measurements falling into the grid. And the map is updated using a one-dimensional Kalman filter. Finally, the elevation of each grid is weighted using a confidence ellipse as output for road elevation estimation.

#### 3.2.1. Grid Height Modeling Based on Light Detection and Ranging Error Model

(1) Noise Characterization of the LiDAR

**Figure 6:**
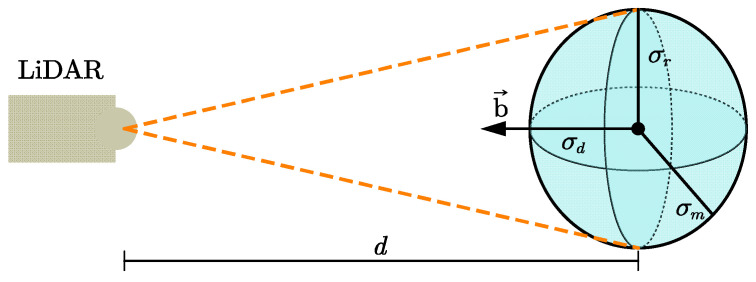
The error model of the LiDAR.

The noise characteristics directly affect the accuracy of the elevation map. The beam model is a commonly used approximation model for LiDAR. However, the complex model results in low efficiency of point cloud data processing. It is difficult to ensure the real-time requirements of elevation map construction. In reference to [35], the LiDAR error model can be represented as an anisotropic model, as shown in Figure 6. The anisotropic error model is parametrized with a vector representing the beam direction $\vec{b}$, supporting the standard deviation on depth $\sigma_{d}$. The standard deviation of the beam $\sigma_{r}$ is supported implicitly by any vector perpendicular to $\vec{b}$. The impact of sunlight, reflection, and large intensity range are ignored. Then, the parameters $\sigma_{r}$ and $\sigma_{d}$ are defined as follows:

(7) $$\sigma_{r}=\frac{0.6d+1.48}{1000}$$

(8) $$\sigma_{d}=0.012$$

In addition, the error model is further simplified as an isotropic representation with only one standard deviation $\sigma_{m}$ and is defined as follows:

(9) $$\sigma_{m}=\max\left\{\sigma_{r},\sigma_{d}\right\}$$

(2) Definition of Grid Height

There are multiple point clouds that fall into one grid, and it is necessary to form a unified description of the grid height through certain processing to improve computational efficiency. After modeling the aforementioned LiDAR error model, each measurement can be described as a Gaussian distribution. Therefore, this section uses maximum likelihood estimation to obtain a unified description of the Gaussian distribution of grid height.

The grid height in the map coordinate system M is defined as a Gaussian distribution $N\left(p,\sigma_{p}^{2}\right)$. And the LiDAR measurements are also approximated as Gaussian distribution $N\left(p_{i},\sigma_{p_{i}}^{2}\right)$ by the error model. When many different measurements $p_{i}$ with known variances $\sigma_{p_{i}}^{2}$ fall into the grid, each grid of the elevation map is updated by multiple LiDAR measurements. The maximum-likelihood estimation [36] is adopted in this paper to calculate the grid height.

The probability density function of a Gaussian distribution with mean p and variance $\sigma_{p_{i}}^{2}$ is described as the following:

(10) $$f_{i}\left(p_{i}|p,\sigma_{p_{i}}^{2}\right)=\frac{1}{\sqrt{2\pi \sigma_{p_{i}}^{2}}}\exp\left(-\frac{{\left(p_{i}-p\right)}^{2}}{2\sigma_{p_{i}}^{2}}\right)$$

The likelihood function L(p) is calculated from the product of the probability density function of all n measurements. The equation is as follows:

(11) $$L\left(p\right)=\prod_{i=1}^{n}\frac{1}{\sqrt{2\pi \sigma_{p_{i}}^{2}}}\exp\left(-\frac{{\left(p_{i}-p\right)}^{2}}{2\sigma_{p_{i}}^{2}}\right)$$

To determine the maximum of the likelihood function, the derivative of the likelihood function is taken, and its derivative is set to zero. Taking the logarithm of the likelihood function simplifies the calculation process, and the result remains the same.

(12) $$\frac{\partial }{\partial p}\log L\left(p\right)=\sum_{i=1}^{n}\frac{p_{i}-p}{\sigma_{p_{i}}^{2}}$$

Let Equation (13) be equal to 0. Then, solving this equation results in the following:

(13) $$p=\sum_{i=1}^{n}\frac{p_{i}}{\sigma_{p_{i}}^{2}}/\sum_{i=1}^{n}\frac{1}{\sigma_{p_{i}}^{2}}$$

(14) $$\sigma_{p}^{2}=\frac{1}{n}\sum_{i=1}^{n}{\left(p_{i}-p\right)}^{2}$$

#### 3.2.2. Map Update from Grid Height

(1) Coordinate Systems

**Figure 7:**
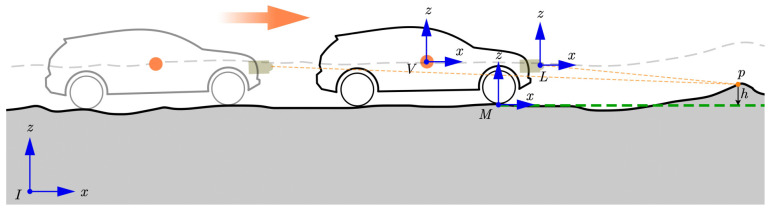
Illustration of the coordinate systems.

As shown in Figure 7, there are four coordinate frames in this paper: the vehicle coordinate system V, the LiDAR coordinate system L, the map coordinate system M, and the internal coordinate system I. The vehicle coordinate system V is fixed to the vehicle centroid (the orange dot in the vehicle). And the LiDAR coordinate system L is fixed to the LiDAR centroid. There exists a known transformation $T_{LV}$ between the LiDAR coordinate system and the vehicle coordinate system V. With this known transformation, we can convert the LiDAR measurements to the vehicle coordinate system. The map coordinate system M is defined in relation to the vehicle coordinate system V with transformation $T_{VM}$. The internal system is fixed to the environment. And it is used as a reference for other coordinate systems. The orange arrow indicates the forward direction of the vehicle. The orange dashed lines describe the LiDAR range measurement for point p.

(2) Elevation Map Update Based on Motion Uncertainty

Map update mainly consists of two parts. One part is to use the grid height modeled in the previous section to achieve elevation map construction through one-dimensional Kalman filtering. The other part is to derive the error propagation of pose estimation in map update.

The Kalman filter is implemented to achieve elevation map estimation $\left(\hat{h},\sigma_{h}^{2}\right)$ from the grid height measurement $\left(p,\sigma_{p}^{2}\right)$. When the LiDAR scans a point *p*, it needs to be mapped to the map coordinate system. As shown in Figure 7, a single measurement, given as the range rLPiL in the LiDAR frame, can be transformed into the corresponding measurement $p_{i}$ with

(15) $$p_{i}=proj_{z}(\Phi_{LM}^{-1}{(}_{L}r_{LP_{i}}){-}_{M}r_{LM})$$

where $proj_{z}=[\begin{array}{ccc} 0 & 0 & 1 \end{array}]$ maps the three-dimensional measurement to the scalar measurement $p_{i}$.

However, the grid height is established in the map coordinate system, while the LiDAR noise model is established in the LiDAR coordinate system. Therefore, it is necessary to calculate the error propagation introduced by coordinate system conversion. The error propagation for the variance $\sigma_{p_{i}}^{2}$ is given as

(16) $$\sigma_{P_{i}}^{2}=J_{L}\Sigma_{L}J_{L}^{T}+J_{\Phi }\Sigma_{\Phi_{IL}}J_{\Phi }^{T}$$

where $J_{L}$ and $J_{\Phi }$ represent the Jacobians for the LiDAR measurement and the sensor frame rotation, respectively. $J_{L}$ and $J_{\Phi }$ are described as

(17) $$J_{L}=\frac{\partial p}{\partial_{L}r_{LP}}=proj_{z}C{\left(\Phi_{LM}\right)}^{T}$$

(18) JΦ=∂p∂ΦLM=projzC(ΦLM)TrLP×L

where C(·) is used to describe the mapping to the corresponding rotation matrix. The superscript × represents the skew-symmetric matrix of the corresponding vector.

Moreover, $\Sigma_{\Phi_{IL}}$ represents the rotation covariance matrix of the LiDAR. According to the relationship defined by the coordinate system, the z-axis of the elevation map coordinate system and the inertial coordinate system are always aligned. And the LiDAR coordinate system and elevation map coordinate system are fixedly connected to the vehicle body. Therefore, the measurement uncertainty of the z-axis rotation in $\Sigma_{\Phi_{IL}}$ is zero. $\Sigma_{\Phi_{IL}}$ only includes uncertainty in pitch and roll. $\Sigma_{L}$ is the covariance matrix of the LiDAR error model. According to the isotropic error model defined above, the equation is as follows:

(19) $$\Sigma_{L}=\left[\begin{array}{ccc} \sigma_{m}^{2} & 0 & 0\\ 0 & \sigma_{m}^{2} & 0\\ 0 & 0 & \sigma_{m}^{2} \end{array}\right]$$

In this paper, we only selected the grid height measurement $\left(p,\sigma_{p}^{2}\right)$ as the state. Due to the irregular changes in the height direction, the one-dimensional Kalman filter only includes the update part.

(20) $$K(k)=\sigma_{h}^{2-}(k)H^{T}{\left(H\sigma_{h}^{2-}(k)H^{T}+\sigma_{p}^{2}(k)\right)}^{-1}$$

(21) $$\hat{h}(k)={\hat{h}}^{-}(k)+K(k)\left(p(k)-H{\hat{h}}^{-}(k)\right)$$

(22) $$\sigma_{h}^{2}(k)=\left(I-K(k)H\right)\sigma_{h}^{2-}(k)$$

where ${\hat{h}}^{-}(k)$ and $\sigma_{h}^{2-}(k)$ represent the priori estimation of the elevation map and its error covariance at time step *k*, respectively. $\hat{h}(k-1)$ and $\sigma_{h}^{2}(k-1)$ represent the state estimation of the elevation map and its error covariance at time step *k*, respectively. p(k) is the grid height measurement at time step *k*. And $\sigma_{p}^{2}(k)$ is the measurement noise covariance at time step *k*. K(k) is the Kalman gain at time step *k*. H(k) is the measurement matrix at time step *k*. I represents the identity matrix. H represents the measurement matrix, which is an identity matrix. Consequently, the one-dimensional Kalman filter is rewritten as the following:

(23) $$\hat{h}(k)=\frac{\sigma_{p}^{2}(k){\hat{h}}^{-}(k)+\sigma_{h}^{2-}(k)p(k)}{\sigma_{p}^{2}(k)+\sigma_{h}^{2-}(k)}$$

(24) $$\sigma_{h}^{2}(k)=\frac{\sigma_{h}^{2-}(k)\sigma_{p}^{2}(k)}{\sigma_{h}^{2-}(k)+\sigma_{p}^{2}(k)}$$

**Figure 8:**
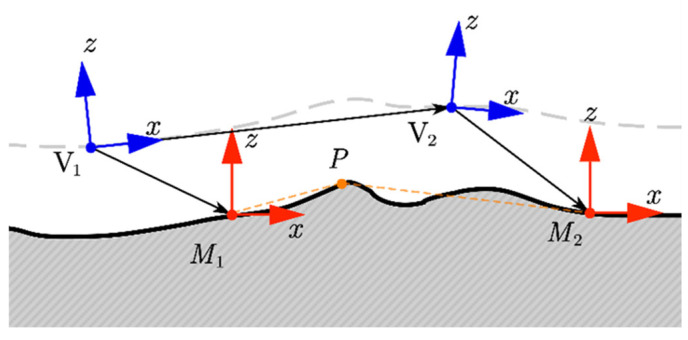
Demonstration of map update based on motion uncertainty.

Due to the assumption that the inertial coordinate system is stationary and the elevation map coordinate system is fixed to the vehicle body, it is necessary to align the map in consecutive frames by pose estimation. As shown in Figure 8, for time *k*, the map coordinate system (blue coordinate system) is associated with the vehicle coordinate system (red coordinate system) through mapping $\left(r_{{\tilde{V}}_{k}M_{k}},\Phi_{{\tilde{V}}_{k}M_{k}}\right)$. At time *k* = 2, the estimation ${\hat{r}}_{M_{2}P}$ of point P in the map coordinate system $M_{2}$ can be represented by the estimation ${\hat{r}}_{M_{1}P}$ of the map coordinate system $M_{1}$ at time *k* = 1 (orange dashed line), i.e., the following:

(25) $${\hat{r}}_{M_{2}P}=-r_{{\tilde{V}}_{2}M_{2}}-{\hat{r}}_{{\tilde{V}}_{1}{\tilde{V}}_{2}}+r_{{\tilde{V}}_{1}M_{1}}+{\hat{r}}_{M_{1}P}$$

Rewrite Equation (24) using the reference coordinate system $M_{2}$ as

(26) rM2PM2=−rV˜2M2−ΦV˜2M2−1(V˜2r^V˜1V˜2)+Φ^M1M2−1(M1rV˜1M1+M1r^M1P)

By using Equation (25), any estimation in $M_{1}$ can be mapped to $M_{2}$. And assuming $r_{M_{k}P}\sim N\left({\hat{r}}_{M_{k}P},\Sigma_{P,k}\right)$, the propagation of covariance from *k* = 1 to *k* = 2 can be written as follows:

(27) $$\Sigma_{P,2}=J_{P}\Sigma_{P,1}J_{P}^{T}+J_{r}\Sigma_{r}J_{r}^{T}+J_{\Phi }\Sigma_{\Phi }J_{\Phi }^{T}$$

where $\Sigma_{P,1}$ is the covariance matrix at *k* = 1. The covariance matrices $\Sigma_{r}$ and $\Sigma_{\Phi }$ represent the motion uncertainty of the vehicle reference coordinate systems ${\tilde{V}}_{1}$ to ${\tilde{V}}_{2}$. They are both modeled by Gaussian distribution, $r_{{\tilde{V}}_{1}{\tilde{V}}_{2}}\sim N\left({\hat{r}}_{{\tilde{V}}_{1}{\tilde{V}}_{2}},\Sigma_{r}\right)$ and $\Phi_{{\tilde{V}}_{1}{\tilde{V}}_{2}}\sim N\left({\hat{\Phi }}_{{\tilde{V}}_{1}{\tilde{V}}_{2}},\Sigma_{\Phi }\right)$. Moreover, the Jacobian matrix corresponding to the three parts is as follows:

(28) $$J_{P}=\frac{\partial_{M_{2}}{\hat{r}}_{M_{2}P}}{\partial_{M_{1}}{\hat{r}}_{M_{1}P}}=C{\left({\hat{\Phi }}_{M_{1}M_{2}}\right)}^{T}=I$$

(29) $$J_{r}=\frac{\partial_{M_{2}}{\hat{r}}_{M_{2}P}}{\partial_{{\tilde{V}}_{2}}{\hat{r}}_{{\tilde{V}}_{1}{\tilde{V}}_{2}}}=-C{\left({\hat{\Phi }}_{{\tilde{V}}_{2}M_{2}}\right)}^{T}$$

(30) $$J_{\Phi }=\frac{\partial_{M_{2}}{\hat{r}}_{M_{2}P}}{\partial {\hat{\Phi }}_{{\tilde{V}}_{1}{\tilde{V}}_{2}}}=-{{(}_{M_{1}}r_{{\tilde{V}}_{1}M_{1}}{+}_{M_{1}}{\hat{r}}_{M_{1}P})}^{\times }C{\left({\hat{\Phi }}_{{\tilde{V}}_{1}M_{1}}\right)}^{T}$$

(3) Elevation Weighted Method Based on Confidence Eclipse

Motion uncertainty not only affects elevation but also accumulates errors in the xy direction. When calculating the height of a grid, the neighboring grids covered by motion uncertainty also have an impact on it. Therefore, this section uses the confidence ellipse method to search a certain range based on covariance to weigh the elevation value of the grid. Only the grid within the area where the wheel trajectory passes through is height-weighted, which can filter the elevation while ensuring real-time performance. We adopted a 95% confidence ellipse to extract grid elevation, including the acquisition of axis vectors and the calculation of fusion weights.

The major and minor axis of the confidence ellipse with a 95% confidence are $2\sqrt{5.991\lambda_{1}}$ and $2\sqrt{5.991\lambda_{2}}$, respectively, where $\lambda_{1}$ and $\lambda_{2}$ are the maximum eigenvalue and minimum eigenvalue of the covariance matrix. If the x-axis and y-axis data have a correlation, then the major and minor axes of the confidence ellipse are not aligned with the coordinate axis. And the angle between them can be determined by the eigenvectors corresponding to the eigenvalues. The angle between the major axis and the x-axis is calculated with the following:

(31) $$\gamma =\arctan\left(\frac{\upsilon_{1}}{\upsilon_{x}}\right)$$

where $\upsilon_{1}$ represents the eigenvector corresponding to the maximum eigenvalue of the covariance matrix, namely, the eigenvector of the major axis. $\upsilon_{x}$ represents the unit vector of the x-axis.

For a grid, assuming its confidence ellipse covers *n* adjacent grids, the weight of the surrounding grid *i* to that grid is $\omega_{i}$, which is defined as the following:

(32) $$\omega_{i}=\left(CDF_{x}(a_{x}+\frac{b}{2})-CDF_{x}(a_{x}-\frac{b}{2})\right)\cdot \left(CDF_{y}(a_{y}+\frac{b}{2})-CDF_{y}(a_{y}-\frac{b}{2})\right)$$

where $a_{x}$ and $a_{y}$ represent the distance from grid *i* to the updating grid in the x and y directions, respectively. $CDF_{x}$ and $CDF_{y}$ represent the Gaussian cumulative distribution function in the x and y directions, respectively.

Finally, the fusion elevation of the grid is calculated after obtaining the weights of all adjacent grids within the confidence ellipse with the following formulas:

(33) $$\hat{h}=\frac{\sum_{i=1}^{n}\omega_{i}{\hat{h}}_{i}}{\sum_{i=1}^{n}\omega_{i}}$$

(34) $$\sigma_{h}^{2}=\frac{\sum_{i=1}^{n}\omega_{i}(\sigma_{h,i}^{2}+{\hat{h}}_{i}^{2})}{\sum_{i=1}^{n}\omega_{i}}-{\hat{h}}^{2}$$

(4) The Gate Strategy Based on Mahalanobis Distance

Since there is no clear kinematics relationship for height change, the map cannot realize the sudden change in height immediately when encountering an object whose height direction changes sharply. Therefore, this section proposed a gate strategy to help the map respond quickly.

The gate based on the Mahalanobis distance is defined as the following:

(35) $$Mahalanobis(k)={\left(p(k)-\hat{h}(k-1)\right)}^{T}{\left(\frac{\sigma_{h}^{2}(k-1)+\sigma_{p}^{2}(k)}{2}\right)}^{-1}\left(p(k)-\hat{h}(k-1)\right)$$

If the Mahalanobis distance is less than gate size *c*, it is considered that the measurement falls into the gate, and there is no sharp change in elevation. When encountering objects with sharp increases or decreases in height, such as walls and stone piers, we selected the larger of the measurement and estimated it as output. Based on the above constraints, the gate strategy is described as follows:

(36) $$\hat{h}(k)=\{\begin{array}{cc} p(k) & p(k)>\hat{h}(k-1)\;and\;Mahalanobis(k)>c\\ \hat{h}(k-1) & p(k)<\hat{h}(k-1)\;and\;Mahalanobis(k)>c\\ \frac{\sigma_{h}^{2}(k-1)p(k)+\sigma_{p}^{2}(k)\hat{h}(k-1)}{\sigma_{h}^{2}(k-1)+\sigma_{p}^{2}(k)} & else \end{array}$$

(37) $$\sigma_{\hat{h}}^{2}(k)=\{\begin{array}{cc} \sigma_{p}^{2}(k) & p(k)>\hat{h}(k-1)\;and\;Mahalanobis(k)>c\\ \sigma_{h}^{2}(k-1) & p(k)<\hat{h}(k-1)\;and\;Mahalanobis(k)>c\\ \frac{1}{\frac{1}{\sigma_{h}^{2}(k-1)}+\frac{1}{\sigma_{p}^{2}(k)}} & else \end{array}$$

## 4. Experiment Evaluation

**Figure 9:**
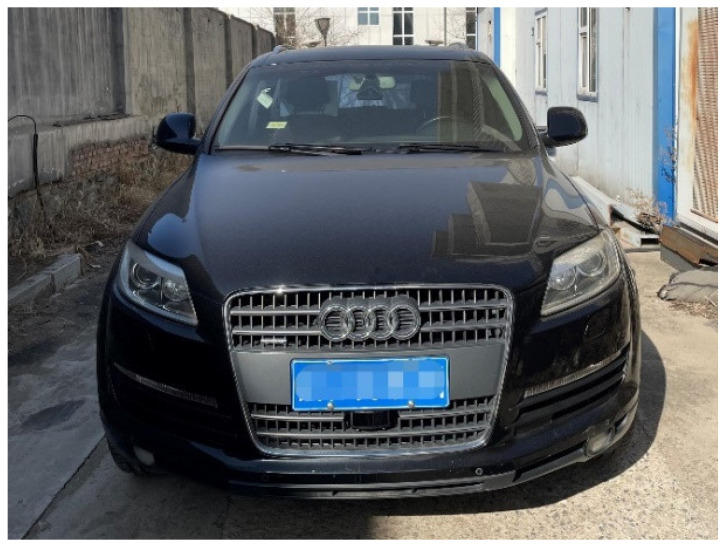
The sport utility vehicle demonstrator.

The sport utility vehicle demonstrator equipped with a MEMS solid-state LiDAR and a GPS/IMU is shown in Figure 9. The sensor parameters are listed in Table 2. The MEMS solid-state LiDAR is installed in the middle bottom of the front of the vehicle, and the GPS/IMU is installed in the center of the rear axle in the trunk. The proposed road elevation estimation algorithm is executed on an IPC ADVANTECH MIC-770 with an Intel i7-9700E CPU at 2.6 GHz, 16 GB RAM, and Ubuntu 18.04 OS. And it is implemented as a C++ library with an interface to the Robot Operating System (ROS) [37]. For efficient data handling and operations, the software builds upon the grid map library [38]. Furthermore, we evaluated our proposed method from height accuracy and time efficiency in the real-world experiment.

### 4.1. Accuracy Analysis

**Figure 10:**
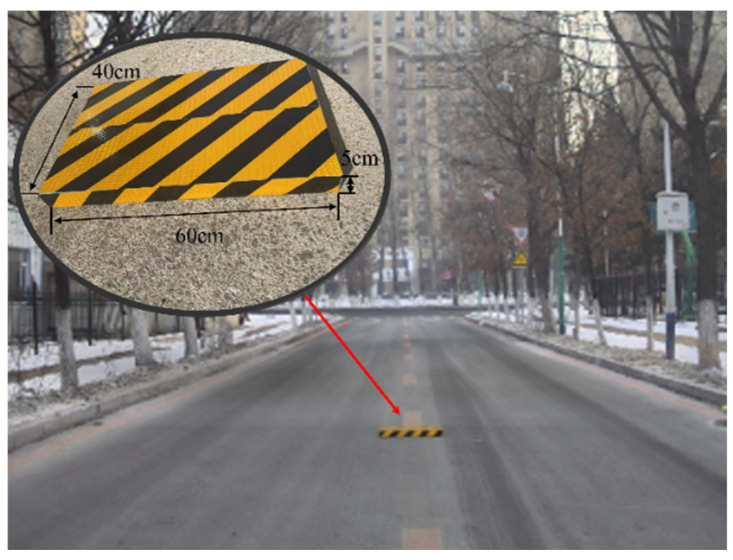
The evaluation scenario.

**Figure 11:**
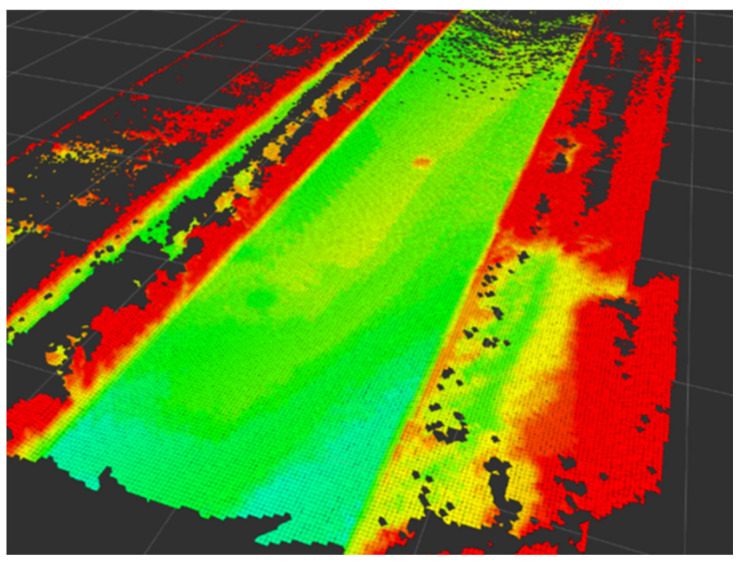
The visualization of road elevation map.

The primary indicator of the mapping algorithm is accuracy since road unevenness is important for ride comfort, which is strongly related to suspension preview control. The evaluation scenario is shown in Figure 10. The car is driven at a speed of 36 km/h. And we put a cuboid with the size of $0.6\times 0.4\times 0.05{\;m}^{3}$ in the scenario. To evaluate the accuracy, the ground truth is divided into two parts. One is the cuboid part, which uses its own size parameters as the ground truth. Another one is the road part, which uses the Hi-Target D8Pro GNSS receiver to survey the road as the ground truth with a position accuracy of 5 mm + 0.5 ppm. The visualization of the road elevation map is shown in Figure 11. In Figure 11, the color of the grid represents the height. And red indicates higher elevation values and green indicates lower elevation values. We manually picked the cuboid from the built map and compared the height with the ground truth, i.e., 0.05 m, to obtain the elevation accuracy. Then, four grid sizes of 0.05, 0.10, 0.15, and 0.20 m are set to evaluate the accuracy with respect to the resolution. Additionally, the Octomap is employed as a comparison, and the results are shown in Table 3. The error of the cuboid height of the proposed method is no more than 5 mm when the resolution is 0.05 m. This error has almost no impact on a tire wheel due to its characteristics [39]. As a comparison, the elevation accuracy is affected by the resolution using Octomap due to the 3D discretization.

### 4.2. Time Efficiency

Time efficiency is another important indicator of the mapping algorithm supporting online control. When the system cannot achieve real-time performance, elevation maps can be sparse and do not sufficiently feed enough information to the controller. We first evaluated the computation time of the elevation map on IPC. Then, we compared the time with other comparative methods to show the evolution in a large-scale environment.

To evaluate the computation time, we ran both methods in the above evaluation scenario and recorded the frame rate. In the proposed method, the map size is $15\times 9{\;m}^{2}$ with 0.05 m resolution. Consequently, our approach achieves almost 25 Hz on IPC and Octomap only 8 Hz. Like most voxel representations, integration of measurements by raycasting and nonrigid transformations is computationally expensive to perform. Therefore, the time required for map generation of Octomap lagged significantly behind that for sensor data acquisition (10 Hz).

**Figure 12:**
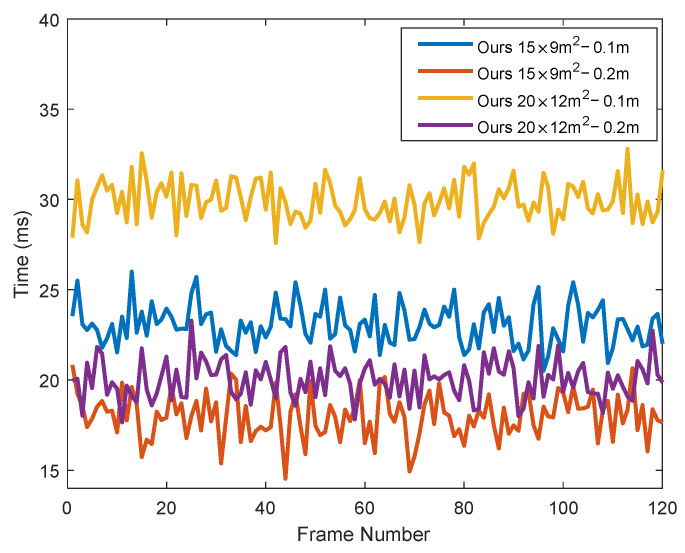
Time scalability of the proposed method.

Moreover, scalability is also an important metric of time efficiency. To validate the scalability, we compared the running time of the elevation map building of the proposed model with the size of $15\times 9{\;m}^{2}$, $20\times 12{\;m}^{2}$, and the resolution of 0.10 m and 0.20 m. As shown in Figure 12, when the size of the elevation map becomes larger, more cells are allocated for map building, resulting in more computation time. Considering that the cells in the map $20\times 12{\;m}^{2}$ with 0.10 m resolution have seven times more grids than those with $15\times 9{\;m}^{2}$ with 0.20 m resolution, the computation time only grows less than one point seven times. In addition, even if the mapping environment becomes larger and larger, the proposed algorithm still has good scalability, which is important for real-time suspension control.

### 4.3. Pose Estimation Performance

**Figure 13:**
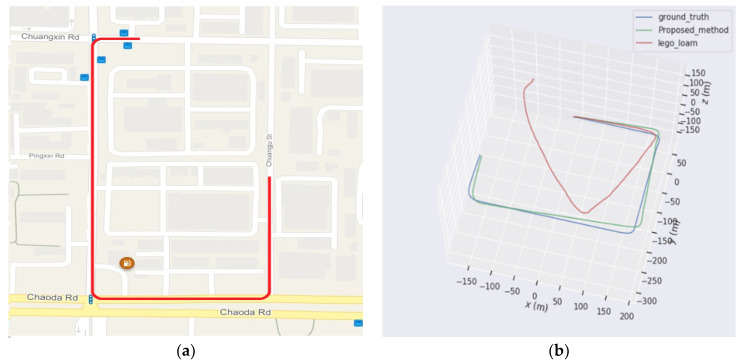

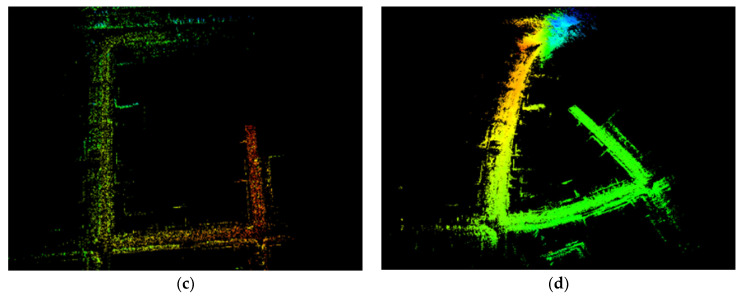
Comparison results of pose estimation algorithm. (**a**) Target trajectory overlaid on Google Maps. (**b**) Estimated trajectories. (**c**) The point clouds aligned with ours. (**d**) The point clouds aligned with LeGO-LOAM.

In the suspension preview control, it is only necessary to extract the elevation from the local map, which has a low requirement for global consistency. However, the generation process of the local map still needs to introduce the vehicle pose. This paper compares our pose estimation algorithm with the open-source algorithm LeGO-LOAM in the urban outdoor scenario that is shown in Figure 13a. The trajectory of the car is the red line in Figure 13a. The car was driven at a speed of 36 km/h. In order to obtain a good GPS signal, the experiment is carried out on an unobstructed road; thus, the feature points may be sparse. In addition, the test is conducted in sunny weather. Moreover, there are some snows on the road that may affect the LiDAR.

The comparison results are shown in Figure 13b, where the trajectories of ground truth, LeGO-LOAM, and our method are plotted in blue, red, and green, respectively. The RTK signal is utilized to represent the ground truth. The proposed method can accurately estimate the trajectory in the urban outdoor scenario. The LeGO-LOAM algorithm can also correctly estimate the vehicle pose when running straight, but the trajectory has a large error during turning. Figure 13c,d are the point clouds aligned by the pose estimated using our proposed algorithm and LeGO-LOAM. It can be shown that the algorithm proposed in this paper has a high consistency, while the LeGO-LOAM algorithm has a poor effect due to the low accuracy of vehicle rotation estimation.

## 5. Conclusions

The traditional method of utilizing suspension sensors makes it hard to handle the irregular road impulses that occur ahead of vehicles in urban environments, which seriously affects ride comfort. Therefore, we have presented a road impulse estimation method in an urban environment with a MEMS LiDAR that addresses the problem of road sensing for suspension preview control. The main novelty of the proposed method is estimating the elevation in the local coordinate so that the process of incorporating the new measurements into the elevation map is only affected by the noise of the range sensor and the accuracy of the pose estimation. The method includes four key components. First, in order to solve the problem of sparse sampling points of the LiDAR, the proposed algorithm deploys a real-time estimation of the vehicle’s 3D pose. Second, taking the sensor error model of LiDAR into account, the proposed algorithm builds the grid height measurement model to aggregate multiple points falling into one grid. Third, it constructs local elevation maps using the Kalman filter and introduces motion uncertainty to accomplish accurate elevation map updates. Lastly, a gate strategy based on the Mahalanobis distance is integrated to deal with the sharp changes in elevation. The proposed method is tested in a real outdoor environment. Finally, the accuracy and efficiency of the proposed method are validated in a real-world experiment, demonstrating its feasibility for road sensing. In the future, it will entail further considering the impact of dynamic objects in the scenario on elevation estimation. Our subsequent work will leverage deep learning for dynamic object detection. Then, they will be separated from the elevation map to overcome any adverse effects of dynamic objects.

## Figures and Tables

**Table 1:** The difference between mechanical and MEMS solid-state LiDAR.

	Type	Frequency	FoV	Horizontal Resolution	Vertical Resolution	Detection Range	Accuracy	Weight
Velodyne HDL-64E	Mechanical	10 Hz	$360^{\circ }\times 26.8^{\circ }$	0.08–0.35°	0.4°	0.5–100 m	2 cm	15.1 kg
RoboSense RS-LiDAR-M1	MEMS Solid-state	10 Hz	$120^{\circ }\times 25^{\circ }$	0.2°	0.2°	0.7–200 m	2 cm	0.8 kg

**Table 2:** The detail parameters of the sensors.

Sensor	Parameters
RoboSense RS-LiDAR-M1	Type: MEMS Solid-state
	Frequency: 10 Hz
	$\mathrm{FoV}:\;120^{\circ }\times 25^{\circ }$
	Horizontal Resolution: 0.2°
	Vertical Resolution: 0.2°
	Weight: 0.8 kg
	Detection Range: 0.7–200 m
	Accuracy: 2 cm
CHC CGI-220	Frequency: 100 Hz
	Position accuracy: 1 cm + 1 ppm (RTK)
	$\mathrm{Pose}\;\mathrm{accuracy}:\;0.1^{\circ }$ (baseline ≥2 m)

**Table 3:** The mapping accuracy comparison.

Resolution (m)	0.01	0.2	0.05	0.10
Ours (cm)	0.48	0.64	0.83	0.97
Octomap (cm)	1.25	2.24	3.59	4.16

## Data Availability

Dataset available on request from the authors.
